# Kidney Function Tests in Health Checkups for Specified Skilled Workers: A Case-based Suggestion

**DOI:** 10.31662/jmaj.2025-0024

**Published:** 2025-08-01

**Authors:** Ami Isoda, Soichiro Saeki, Daisuke Katagiri, Tatsuya Kikuchi, Naho Matsubara, Yuri Katayama, Harui Bamba, Hideki Takano, Chihaya Hinohara

**Affiliations:** 1 Department of Nephrology, National Center for Global Health and Medicine, Japan Institute for Health Security, Tokyo, Japan; 2 Department of Emergency Medicine and Critical Care, National Center for Global Health and Medicine, Japan Institute for Health Security, Tokyo, Japan; 3 International Health Care Center, National Center for Global Health and Medicine, Japan Institute for Health Security, Tokyo, Japan

**Keywords:** CKD, SSW, eGFR

## Abstract

Approximately 250,000 foreign nationals reside in Japan under the specified skilled worker (SSW) visa category. Due to Japan’s aging population and declining birth rate, a labor shortage is anticipated, prompting an increase in the intake of SSWs. All SSWs are required to undergo pre-arrival health screenings, but renal function assessments, such as the estimated glomerular filtration rate (eGFR), are not currently included.

We present a case of a woman in her early 20s from Myanmar who developed severe anemia and renal dysfunction after arriving in Japan as an SSW. Before arrival, an initial health checkup identified anemia with a hemoglobin level of 10.3 g/dL, but renal function tests were not conducted. Ten months later, her hemoglobin had dropped to 7.7 g/dL, and her serum creatinine level was elevated to 7.83 mg/dL. Subsequent testing revealed severe renal dysfunction, and imaging showed extensive cysts in the right kidney and atrophy of the left kidney, suggesting a congenital malformation. She required immediate hemodialysis.

This case highlights the gap in SSW health screenings, where renal function tests are omitted. Given Japan’s rising number of SSWs, we emphasize the importance of including kidney function assessments, such as eGFR, in pre-employment health checkups. Early detection could prevent delays in diagnosing conditions like chronic kidney disease. As hemodialysis initiation often complicates treatment continuity after returning home, appropriate screening is essential for patient safety and improved health outcomes.

In conclusion, we suggest updating the pre-employment health checkup protocol for SSWs to include kidney function tests to address this critical gap.

Approximately 250,000 foreign nationals reside in Japan under the specified skilled worker (SSW) visa category ^(1)^. With a rapidly aging population and declining birthrate, Japan faces a serious labor shortage ^(2)^, prompting an expanded intake of SSWs to improve Japan’s international competitiveness and bolster economic productivity ^(3)^. All SSWs must undergo pre-arrival health examinations ^(1)^; however, these do not currently include renal function assessments such as the estimated glomerular filtration rate (eGFR).

We describe a case of a woman in her early 20s from Myanmar who presented to our hospital with severe anemia and kidney dysfunction after arriving in Japan as an SSW. She had no notable past medical history and had never undergone a health checkup in her home country. Before arriving in Japan as an SSW, an initial health checkup revealed anemia with a hemoglobin level of 10.3 g/dL. Urine protein and occult blood test results were negative. Kidney function tests, such as measuring serum creatinine (sCre) and blood urea nitrogen (BUN) levels, were not conducted.

Approximately 10 months later, during a routine health checkup, her hemoglobin level further declined to 7.7 g/dL, and her sCre level was elevated at 7.83 mg/dL, indicating severe renal dysfunction. The patient was promptly referred to our hospital for further evaluation. Laboratory tests revealed BUN and sCre levels of 102.0 mg/dL and 9.28 mg/dL, respectively. Her eGFR was 5.3 mL/min/1.73 m^2^. Computed tomography revealed that multiple cysts had almost completely replaced her right kidney, whereas her left kidney was markedly atrophic (Figure 1A, B), suggesting a congenital malformation. Hemodialysis was immediately initiated (patient data directly before the initiation of hemodialysis available as Supplementary Figure S1). Although the exact cause of her renal disease remains unclear, her clinical course following dialysis initiation strongly indicated the irreversibility of end-stage kidney disease. She subsequently returned to Myanmar and continued maintenance hemodialysis three times per week.

**Figure 1.**
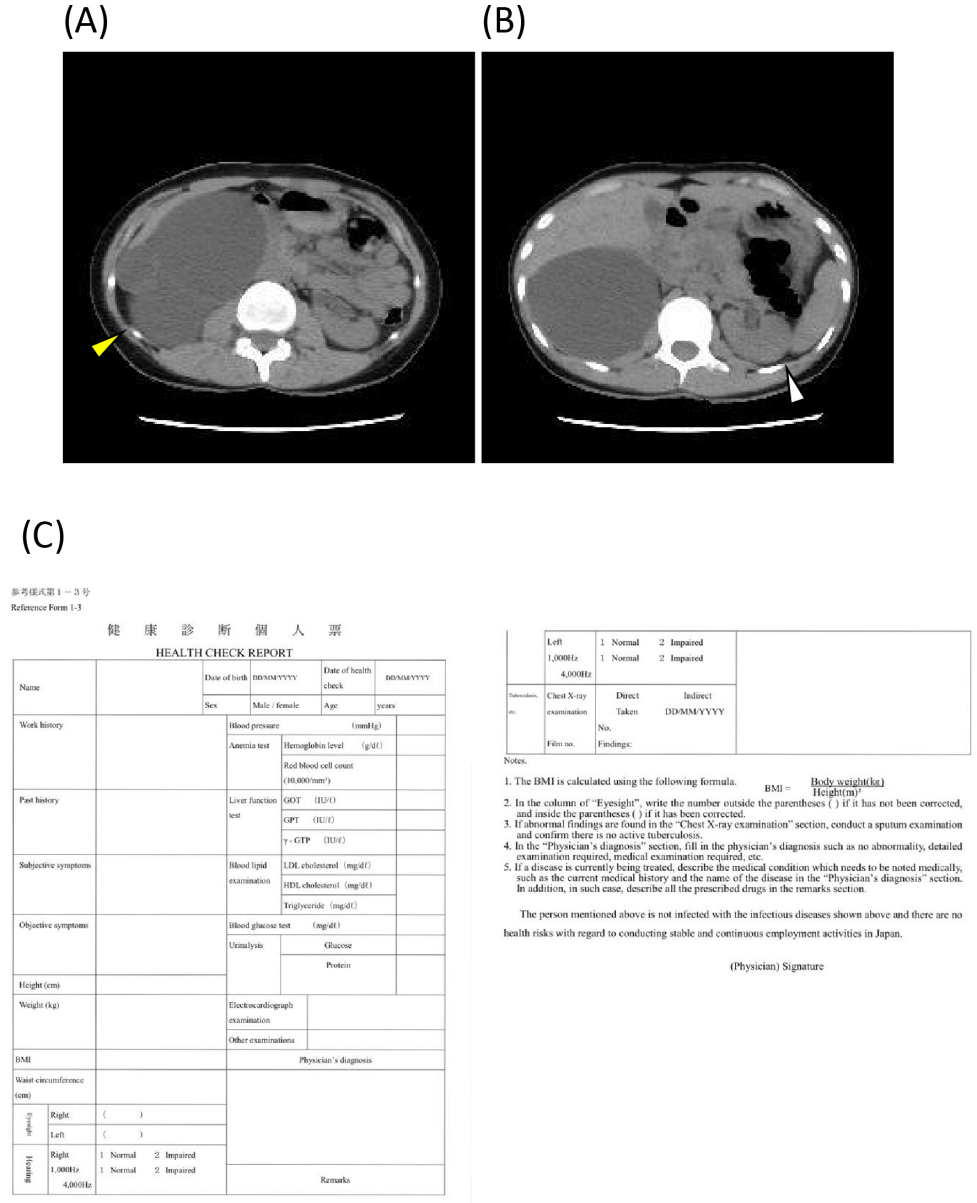
CT findings and health checkup for SSWs. A, B: CT scans of the kidneys. The computed tomography scan shows multiple cysts completely replacing the right kidney. A: yellow arrowhead; significant atrophy in the left kidney. B: white arrowhead. C: Health checkup individual card for SSWs. The card includes mandatory examinations such as chest radiography, physical examinations (height, weight, vision, hearing), blood pressure measurements, blood tests (complete blood count, liver function tests), and urinalysis ^(1)^. CT: computed tomography; SSW: specified skilled worker.

This case highlights a critical gap in the current health checkup requirements for SSWs. Although anemia was identified during the initial screening, the omission of renal function assessment delayed the diagnosis of chronic kidney disease. The pre-employment health checkups for SSWs included chest radiography, physical examination (height, weight, vision, hearing), blood pressure measurements, blood tests (complete blood count, liver function tests), and urinalysis. However, kidney function tests such as eGFR were not included (Figure 1C) ^(4)^.

Although GFR evaluation using the estimation formula (eGFR) is not yet universally practiced worldwide, sCre levels are routinely measured, and the use of the estimation formula is a common approach for the initial assessment of sCre and GFR levels ^(5)^. Unlike Japan’s current SSW health screening, some countries incorporate kidney function tests in their immigrant health screenings, such as sCre measurement in Canada ^(6)^ and both sCre and eGFR assessments in Australia ^(7)^. The number of SSWs is increasing in Japan ^(1)^, and it is essential to recognize potential cases in this population similar to those presented here. Once hemodialysis has been initiated, it is often challenging for individuals to return to their home country and continue treatment smoothly. Therefore, waterfront measures are critical for the safety of both patients and medical institutions.

According to the medical fee points under the Ministry of Health, Labour and Welfare, the cost of adding sCre and eGFR to routine blood tests would be only 160 yen per person. In contrast, while the incidence is much lower, maintenance hemodialysis costs an estimated 40,000 to 50,000 yen per session and must be performed three times weekly indefinitely. This ongoing treatment places a substantial financial burden on patients. Although SSWs are eligible for Japan’s national health insurance ^(8)^, which typically covers 70% of medical costs, patients on dialysis may obtain a Grade 1 Physical Disability Certificate for additional financial support.

In conclusion, we suggest updating the pre-employment health checkup protocol for SSWs to include kidney function tests to address this critical gap and support better health outcomes in this growing population. This small addition could have significant public health and economic benefits for an increasingly essential workforce.

## Article Information

### Conflicts of Interest

None

### Acknowledgement

We thank the Department of Nephrology and International Health Care Center, National Center for Global Health and Medicine, for their support.

### Informed Consent

Informed consent was obtained from the patient included in the report.

## Supplement

Supplementary Figure S1
