# Supplementary material for: Kidney Function Tests in Health Checkups for Specified Skilled Workers: A Case-based Suggestion
Source: JMA J. 2025 Aug 1;8(4):1392–5. doi: 10.31662/jmaj.2025-0024 (PMC12598293; doi:10.31662/jmaj.2025-0024)
Supplement: Supplementary Figure S1 [file 2433-3298-8-4-1392-s001.pdf]

(A)

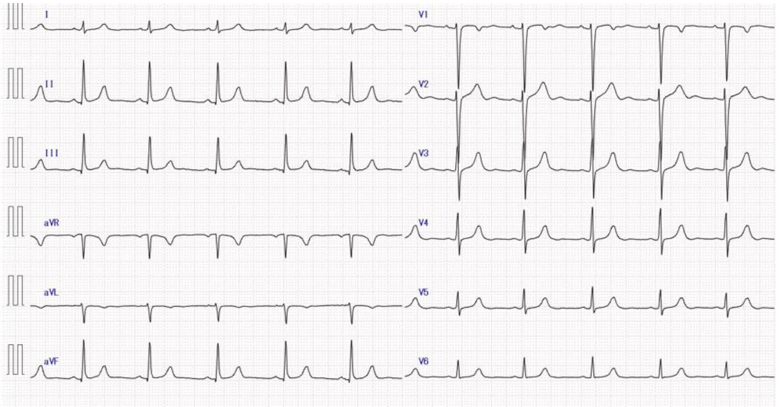

(B)

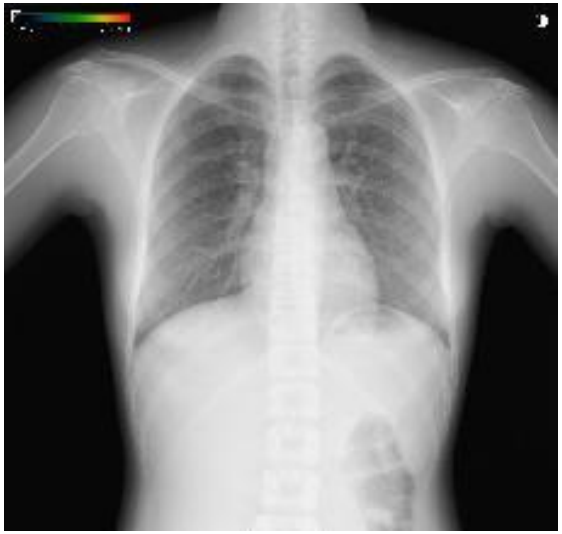

(C)

pH	7.2333	
pCo2	40.5	mmHg
pO2	17.6	mmHg
HCo3-	17.1	mmol/L
tCO2	18.3	mmol/L
Na+	140	mmol/L
K+	4.5	mmol/L
Ca2+	1.10	mmol/L
Hct	23.9	%
Cl-	108	mmol/L
Lac	0.4	mmol/L
tHb	7.8	g/dl
FO2Hb	24.9	%
FCoHb	<1.0	%
FHHb	73.1	%
FMetHb	1.1	%
AnionGap	14.5	mmol/L
Glu	101	mg/dl
tBil	0.6	mg/dl
tO2	2.8	Vol%
p50	27.25	mmHg

Supplementary Figure S1. Patient data directly before the initiation of hemodialysis.
(A) Electrocardiogram (ECG). The ECG shows no significant abnormalities. (B) Chest X-ray. The radiograph shows no obvious infiltrative shadow or consolidation. (C) Venous blood gas analysis. The results include pH, pCO_2, pO_2, HCO_3^-, lactate, and serum electrolyte levels (Na^+, K^+, Cl^-, etc.).
